# Quantification of pulmonary involvement in COVID-19 pneumonia: an upgrade of the *LungQuant* software for lung CT segmentation

**DOI:** 10.1140/epjp/s13360-023-03896-4

**Published:** 2023-04-11

**Authors:** Francesca Lizzi, Ian Postuma, Francesca Brero, Raffaella Fiamma Cabini, Maria Evelina Fantacci, Alessandro Lascialfari, Piernicola Oliva, Lisa Rinaldi, Alessandra Retico

**Affiliations:** 1 Pisa Division, National Institute for Nuclear Physics (INFN), Pisa, Italy; 2 Pavia Division, INFN, Pavia, Italy; 3 Department of Physics, University of Pavia, Pavia, Italy; 4 Department of Mathematics, University of Pavia, Pavia, Italy; 5 Department of Physics, University of Pisa, Pisa, Italy; 6 Department of Chemical, Physical, Mathematical and Natural Sciences, University of Sassari, Sassari, Italy; 7 Cagliari Division, INFN, Cagliari, Italy

## Abstract

Computed tomography (CT) scans are used to evaluate the severity of lung involvement in patients affected by COVID-19 pneumonia. Here, we present an improved version of the *LungQuant* automatic segmentation software (*LungQuant* *v*2), which implements a cascade of three deep neural networks (DNNs) to segment the lungs and the lung lesions associated with COVID-19 pneumonia. The first network (BB-net) defines a bounding box enclosing the lungs, the second one (U-net$_1$) outputs the mask of the lungs, and the final one (U-net$_2$) generates the mask of the COVID-19 lesions. With respect to the previous version (*LungQuant* *v*1), three main improvements are introduced: the BB-net, a new term in the loss function in the U-net for lesion segmentation and a post-processing procedure to separate the right and left lungs. The three DNNs were optimized, trained and tested on publicly available CT scans. We evaluated the system segmentation capability on an independent test set consisting of ten fully annotated CT scans, the COVID-19-CT-Seg benchmark dataset. The test performances are reported by means of the volumetric dice similarity coefficient (vDSC) and the surface dice similarity coefficient (sDSC) between the reference and the segmented objects. *LungQuant* *v*2 achieves a vDSC (sDSC) equal to 0.96 ± 0.01 (0.97 ± 0.01) and 0.69 ± 0.08 (0.83 ± 0.07) for the lung and lesion segmentations, respectively. The output of the segmentation software was then used to assess the percentage of infected lungs, obtaining a Mean Absolute Error (MAE) equal to 2%.

## Introduction

Acute respiratory distress syndrome (ARDS) caused by COVID-19 is the main cause of Intensive Care Unit (ICU) admission and fatality for affected patients [1, 2]. Evaluation of the severity of pulmonary involvement can be performed through computed tomography (CT) images [3–5]. Evaluating CT scans is a time-consuming task; for this reason, artificial intelligence (AI) methods for automated analysis of CT scans can be a useful tool to support the work of physicians [6]. The aim of this kind of approach is to assist the clinician in making an automated assessment of the alteration of the lung parenchyma related to COVID-19 pneumonia. To this purpose, a standardized assessment scheme for the reporting of radiological findings in chest CT of subjects suspected of COVID-19 has been defined [7]. It is based on a five-level scale of increasing suspicion of pulmonary involvement. Another scoring system, directly based on the extent of lung involvement (abnormal lung parenchyma), is the CT severity score (CT-SS), which has been demonstrated to be directly correlated with disease severity [8]. The CT-SS is a score made of five classes which are defined on the basis of the ratio between the volume of the infected areas and the lung one (CT-SS = 1 for *P* < 5%, CT-SS = 2 for 5% $\le$* P* < 25%, CT-SS = 3 for 25% $\le$* P* < 50%, CT-SS = 4 for 50% $\le$* P* < 75%, and CT-SS = 5 for* P* $\ge$ 75%).

The mere visual assessment of lung CT can hardly provide a reliable and reproducible estimate of the percentage of lung involvement. To facilitate this task, an AI-based support tool is highly desirable. The quantification problem that needs to be solved is actually a segmentation problem. To estimate the percentage of the affected lung in COVID-19 pneumonia, it is necessary to accurately segment both the subject’s lungs and the COVID-19-related lesions.

The task of lung segmentation has been addressed over the years with several different techniques, including grey-value thresholding, region growing, isosurface triangulation, morphological operations, and combinations of them [9–13]. However, most traditional approaches fail when abnormalities introduce changes in the normal lung density [14], especially in the specific case where abnormalities are adjacent to the pleura surface. The latter is just the case of most CT images of subjects with COVID-19 lesions. Traditional medical image segmentation methods have gradually given way to data-driven approaches mainly based on machine learning (ML) and deep learning (DL) in the specific field of thoracic imaging [15] and in medical image analysis in general [16]. U-nets [17] are currently outperforming other artificial intelligence (AI)-based methods in the image segmentation task in many research fields. They are also becoming widespread in medical imaging to identify organs, lesions and other regions of interest across several imaging modalities [18, 19]. The main drawback of DL approaches to image segmentation is their need of large annotated datasets for training the models. Collecting data and reliable annotations is particularly difficult and time-consuming especially for image segmentation tasks, where pixel/voxel-level ground truth is required. DL-based lung segmentation approaches demonstrated to be efficient in the accurate identification of lung parenchyma even in case of compromised lung appearance due to COVID-19 infection [20], or to chronic obstructive pulmonary disease (COPD) [21], or to any routine clinical condition affecting the lungs [22]. The challenging task of lung lobe segmentation is tackled in the paper by Xie et al. [20], where the transfer learning of a model trained on thousands of subjects with COPD was applied on a sample of hundreds of subjects affected by COVID-19 pneumonia. Lobe segmentation reference was acquired for all subjects, as it is a fundamental information for model train, test and evaluation. Such large and annotated data samples are not publicly available at present. Since the outbreak of the pandemic, many research groups have developed AI-based approaches to identify and segment ground-glass opacifications (GGO) caused by COVID-19 [23–29].

**Fig. 1:**
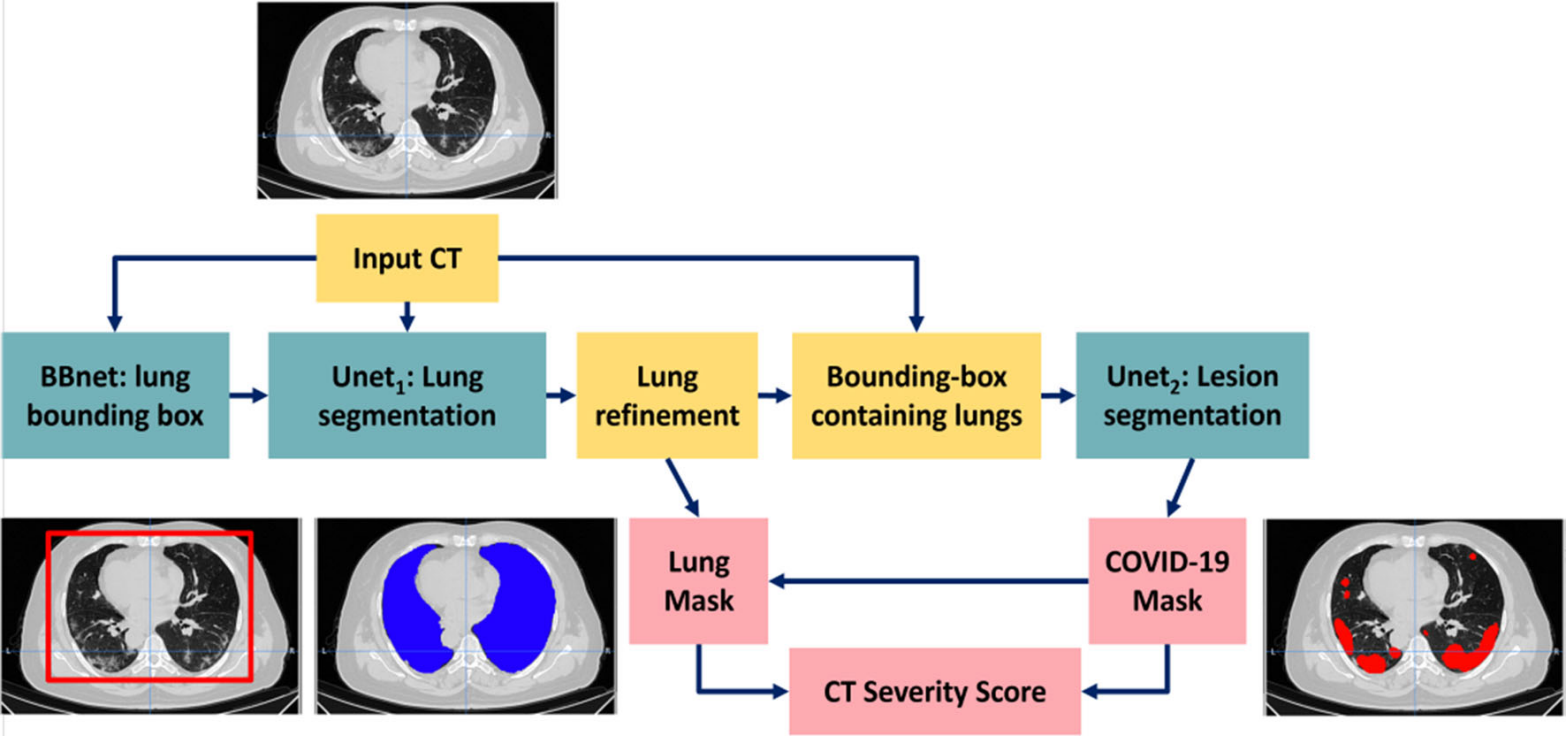
A sketch of the *LungQuant v2* analysis pipeline: the input CT scans are processed by the BB-net, which identifies a bounding box enclosing the lungs to be used to crop the images to be provided in input to U-net$_1$, which is devoted to lung segmentation; its output is refined by a morphology-based method (for details, see reference [30]); a bounding box enclosing the segmented lungs is identified and used to crop the original CT scan to be then processed by U-net$_2$, which is devoted to COVID-19 lesion segmentation. The *LungQuant v2* provides as output: the COVID-19 lesion mask (directly provided by U-net$_2$), the lung mask (which is obtained as the logical union between the outputs of U-net$_1$ and U-net$_2$), and the ratio between the COVID-19 lesion and the lung volumes, which provides the percentage of affected lung volume and the CT-SS for each patient

In this work, we present an improvement of the *LungQuant* *v*1 algorithm we have previously developed for the segmentation and quantification of the lung volume affected by COVID-19 lesions [30]. The new software version we propose, the *LungQuant* *v*2 publicly released package,^1^ is composed of three sequential deep neural networks (DNNs), as sketched in Fig. 1. The first DNN, which is referred to as BB-net, identifies two points on a CT image, $(x_1, y_1, z_1)$ and $(x_2, y_2, z_2)$, which define the bounding box (BB) enclosing the lungs. This bounding box is then used to crop the CT image to the lung volume. The cropped image is then fed into a second DNN, the U-net$_1$, which outputs the segmentation of the lungs. A third DNN, the U-net$_2$, is then used to segment the lesions related to COVID-19 within the lung. The improvements with respect to the previous version are: (1) the introduction of the DNN for the bounding box, (2) the addition of a post-processing module for the separation of right and left lungs and (3) the introduction of an additive term to the loss function of the last DNN to make the system response more linear with respect to the severity of the lung infection.

The BB-net has been introduced to improve the robustness of the previous version of our analysis pipeline [30]. In this way, the segmentation algorithm can be efficiently used to analyse lung CT images acquired with different field of views (FOV). In fact, it may happen that the CT scan is reconstructed with a smaller FOV than the acquisition one in order to obtain an enlarged image in the region of interest.

## Materials and methods

The process of training a DNN-based segmentation algorithm involves the selection of appropriate training data and tailoring of the network structures to the available data. As for the training of *LungQuant v1* [30] segmentation algorithm, also in this work, public available data samples were used. A summary of the data used in this work is shown in Table 1. The three DNNs used to build the *LungQuant v2* CT analysis pipeline are described in the next sections.

**Table 1:** A summary of the datasets used in this study. The table reports the number of cases available, the availability of: Lung masks, COVID-19 lesions masks and CT severity score (CT-SS)

Dataset name	No. of cases	Lung mask	Lesions mask	CT-SS
Plethora [31]	402	Yes	No	No
Lung CT segmentation challenge [32]	60	Yes	No	No
COVID-19 challenge [33]	199	No	Yes	No
MosMed [34]	1110	Only 91 (in-house made)	Only 50	Inferable
COVID-19-CT-Seg [29]	10	Yes	Yes	Inferable

### DNN for lung bounding box regression: the BB-net

**Fig. 2:**
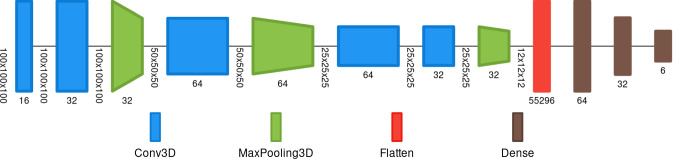
Graphical representation of the BB-net, image obtained with Net2Vis software [36]

The network model we chose for selecting the lung bounding box (BB-net) is based on the AlexNet [35]. Figure 2 shows a graphical representation of the model. The input image has a shape of $100 \times 100 \times 100$ voxels, which is obtained by resampling the original CT scan. Then, the image is windowed in the Hounsfield unit range [−1000, 1000], and then linearly scaled to the [0,1] range. As shown in Fig. 2, the model is made up of a series of convolution, max pooling, flattening and dense layers. The final layer of BB-net is a vector with shape 6 which represents the (x,y,z) coordinates of the two points that define the bounding box enclosing the lungs. The first three values are the first (x,y,z) coordinates, and the latter three, the second point. The training was performed through a regression, the loss value being the Mean Square Error (MSE), which computes the distance between the true bounding box (defined by two points) and the predicted box.

**Fig. 3:**
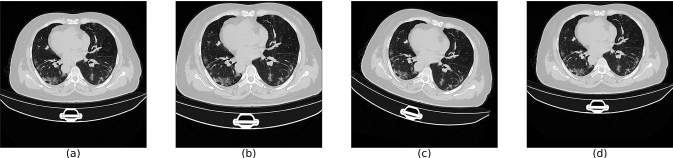
Example of the data augmentation performed to generate the BB-net training set: **a** Image without data augmentation; **b** Zooming to reduce the FOV; **c** Rotation; **d** Shift

BB-net was trained on the data shown in Table 1 for which lung masks were available to derive reference bounding boxes for model training. Since the data set is small, not all the available inputs are well-represented. In particular, there is an unbalance in the different image FOVs. Most of the publicly available CT scans have large FOVs, and a very limited amount of CT scans showed a FOV more focused over the lung volume. For this reason data augmentation was implemented by reducing the FOV, rotating and displacing the centre of the images. An example of these augmentation techniques is provided in Fig. 3.

The training of the network was performed in two steps. Firstly, we optimized the following hyper-parameters (as described below):

- *Kernel size*, Modifying the initial convolution kernel size with values from this list: (4, 8, 16, 32, 64, 128), keeping the ratio of the convolution kernels as shown in Fig. 2.
- *Dense layer size*, Modifying the second-last dense layer size with values from this list: (4, 8, 16, 32), while the first dense layer being double the size of the modified one.
- *Optimizer*, The optimizer changed between Adam, Adagrad and RMSprop.
- *Learning rate*, The tested initial learning rate values were: 10$^{-2}$, 10$^{-3}$, 10$^{-4}$.

This task was performed by training the BB-net on 80% of the available data (i.e. Plethora, Lung CT Segmentation Challenge, COVID-19 Challenge and MosMed) and its augmentation, while leaving 10% as validation data and 10% as test data. The latter 20% of data was composed only by the original data, i.e. without augmentation. Once the hyper-parameters were optimized, the final training of the BB-net was performed on the same train, test and validation. The weights which provided the lowest loss value on the validation set were saved and stored.

### U-net$_1$ and U-net$_2$ for lung and lesion segmentation

The segmentation DNN model we chose is a U-net [17] which is a standard fully convolutional neural network, particularly suited for segmentation tasks. With regard to the U-net for lung segmentation (U-net$_1$), the architecture and the training parameters are the same as in *LungQuant v1*, and are fully detailed in Lizzi et al. [30]. U-net$_1$ has been trained to segment the lungs relying on the available data with lung annotations described in Table 1, mainly composed by patients not affected by SARS-CoV-2 pneumonia. The difference with respect to the *LungQuant v1* release consists in the pre-processing of the CT scans. In fact, in order to reduce the variability of the performance due to the different FOVs, the images have been cropped to the bounding box inferred by the BB-net trained to this scope. The U-net for lung segmentation is thus trained on image portions (resampled to the U-net input size), where the lungs appear with a standardized size, thus facilitating the learning process.

We introduced in the *LungQuant v2* segmentation pipeline a post-processing script to separate the left and right lung, which is based on a watershed transformation. The separation between right and left lungs is not trivial on CT scans since it is not unusual that lungs appear as if they were connected especially near to the neck. Once the system computes the lung segmentation, the mask is firstly resized at half of its initial size. This was necessary to reduce the computing time of the following procedure. Then, the Euclidean distance transform is applied to the resized lung mask as well as a Gaussian filter to reduce noise. Using the peak_local_max function of scikit-image, the local maxima has been computed on the Euclidean distance and hence applied the watershed segmentation.

The lesion segmentation, instead, has been made using U-net$_2$ that has slightly changed with respect to the one integrated in the *LungQuant v1* software, due to the fact that a different loss function has been introduced. In fact, since *LungQuant* *v*1 underestimates the most severe cases, we applied a strategy to make the algorithm response more linear with severity. This defect was mainly due to the unbalanced data used to train the lesion segmentation. In fact, the available public datasets mainly contain cases with mild severity. Moreover, it is not straightforward to design a data augmentation strategy to augment severe cases only. For this reason, a different loss function has been defined to re-train the U-net$_2$. The vDSC used to train the U-net$_2$ of *LungQuant v1* in fact is a volume metric which inflates when the volumes to be segmented are small. For this reason, a new term has been added to the loss function, and it is defined as follows:

1 $$\begin{aligned} L_s = \sum _{x \in \Omega } F_{\rm pred} \cdot (B_{\rm true} - F_{\rm true}) \end{aligned}$$

where $F_{\rm pred}$ and $F_{\rm true}$ are the predicted and the reference foreground masks respectively and $B_{\rm true}$ is the reference background mask. The loss function we used is hence defined as follows:

2 $$\begin{aligned}{} & {} {L} = {\rm vDSC}_{\rm loss} + CE_{\rm weighted} + L_s \end{aligned}$$

3 $$\begin{aligned}{} & {} {\rm CE}_{\rm weighted} = w(x) \sum _{x \in \Omega } log (M_{\rm true}(x) \cdot M_{\rm pred}(x)) \end{aligned}$$

where CE stands for cross entropy, *w*(*x*) is the weight map which takes into account the frequency of white voxels, *x* is the current sample and $\Omega$ is the training set. The U-net$_2$ devoted to lesion segmentation has been trained for 150 epochs, and the training has been stopped at the best validation vDSC. The performance of the *LungQuant*2 has been evaluated on the external independent data set COVID-19-CT-Seg.

## Results

The BB-net training results are provided on the inner test set. Then, the performance obtained by the overall segmentation system, the *LungQuant* *v*2 pipeline are provided on the completely independent test set, the COVID-19-CT-Seg dataset [29], consisting of ten fully annotated CT scans that can be used as a benchmark.

### BB-net performance

**Fig. 4:**
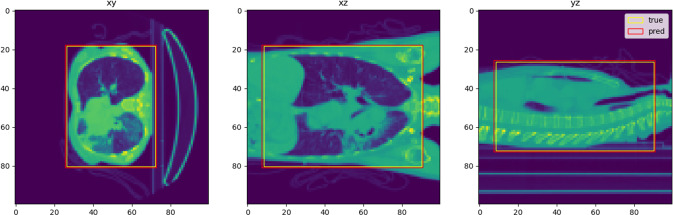
BB-net: a predicted bounding box example (red rectangle), compared to the true bounding box (yellow rectangle)

We optimized the hyper-parameters of the BB-net with a grid search, and the most performing one is represented in Fig. 2. The training loss reaches a plateau value which is less than 10$^{-5}$. A typical example of the bounding box around the lung predicted by the BB-net is shown in Fig. 4. The red square inside the image shows the predicted bounding box, which nearly perfectly overlaps the true bounding box (yellow square), is obtained from the reference lung masks of the annotated CT scans.

### Results on the *LungQuant v2* pipeline on the COVID-19-CT-Seg benchmark dataset

**Fig. 5:**
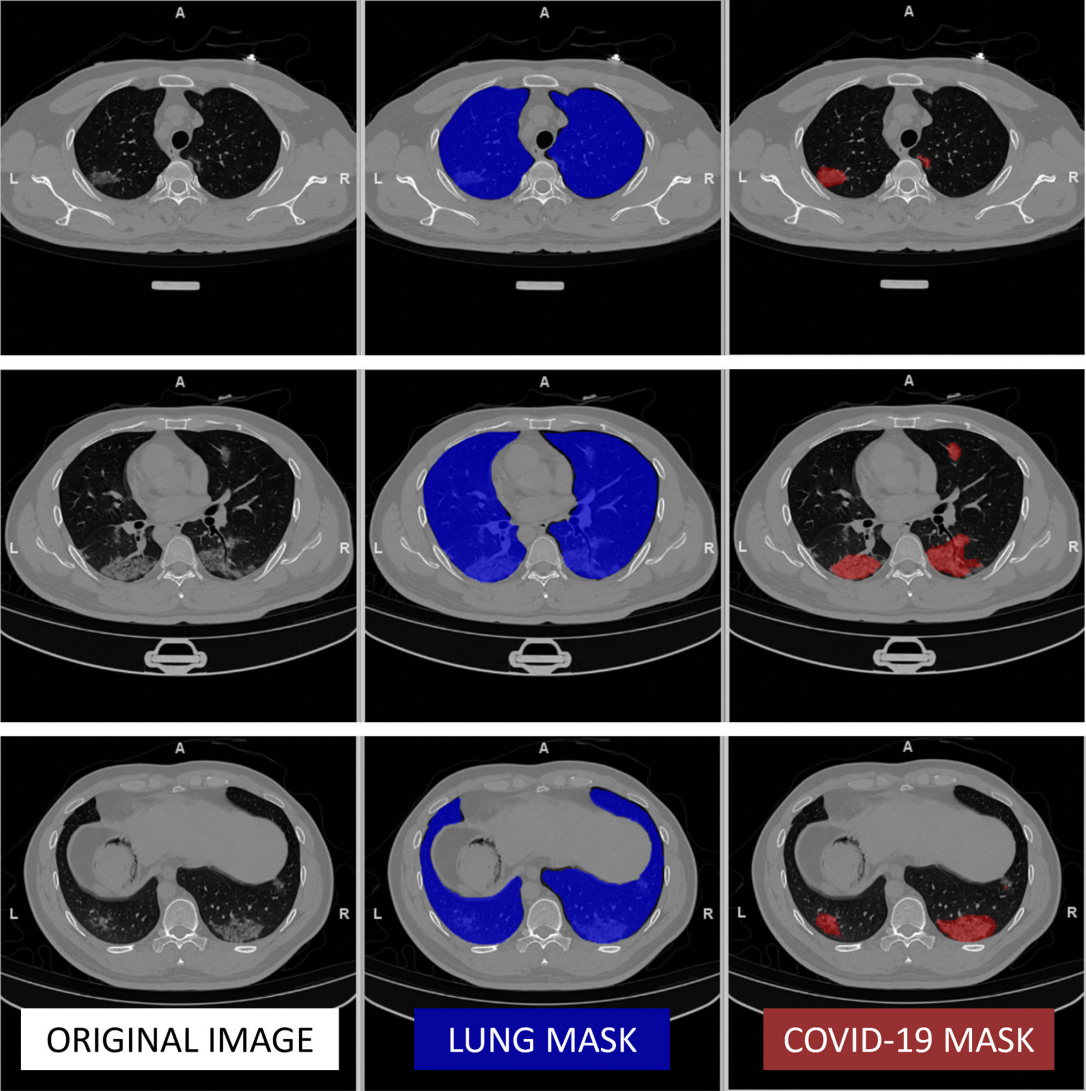
*LungQuant* *v*2 system: axial slices of case coronacases008.nii from COVID-19-CT-Seg test dataset. On the columns: original images (left), predicted lung (centre) and COVID-19 lesion masks (right)

**Fig. 6:**
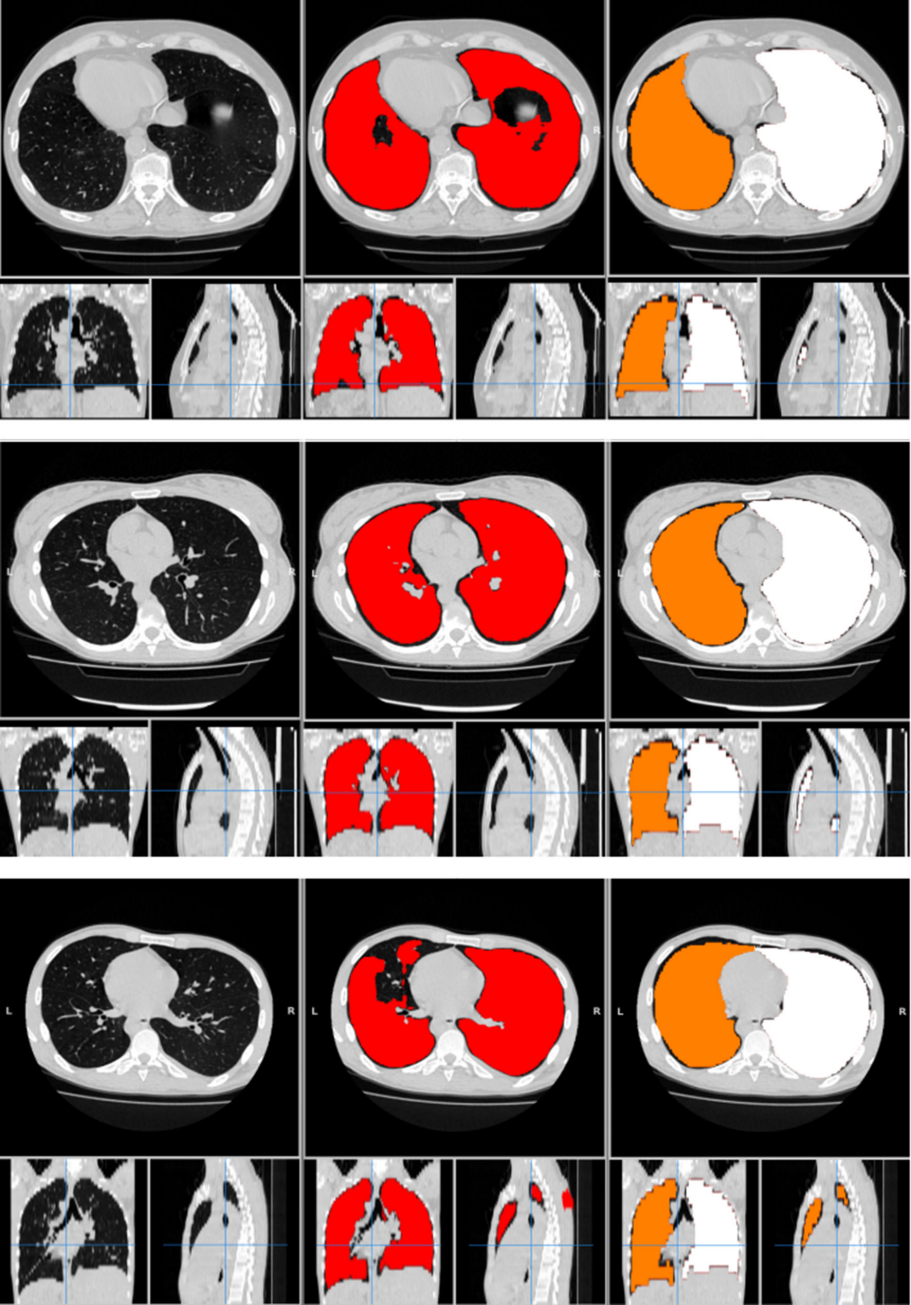
Visual assessment of the lung segmentations made with *LungQuant* *v*1 and *LungQuant* *v*2. On the left: the original image (cases study0002.nii, study0089.nii and study1064.nii from MosMed dataset). On the centre: lung segmentation made by *LungQuant* *v*1. On the right: lung segmentation made by *LungQuant* *v*2

For both U-net$_1$, which is devoted to lung segmentation, and U-net$_2$, which is used to segment the lesions, the volumetric dice similarity coefficient (vDSC) and the surface dice similarity coefficient (sDSC) at 5 mm of tolerance have been computed on the independent test set COVID-19-CT-Seg. The segmentation performances were evaluated separately for the lungs and the lesions. The results of *LungQuant v2* and the comparison with the previous version are reported in Table 2. In Fig. 5, we reported the segmentation outputs computed on a test case (*coronacases008.nii*). Despite the effect on the metrics is limited, the advantage of having introduced the BB-net in the *LungQuant* software is apparent when looking at the segmentations obtained for images with a different FOV, as shown in Fig. 6, where the outputs of *LungQuant v1* and *LungQuant v2* are visually compared on three cases of the MosMed data set. A direct quantitative comparison on labelled images with different FOVs could not be performed, as, to the best of our knowledge, a suitable datasets to be used to this purpose is not publicly available. However, the visual assessment of the two outputs supports the initial intuition that the introduction of the BB-net has a positive effect on the lung segmentation.

**Table 2:** Performances of lung and COVID-19 lesion segmentations made by *LungQuant v1* and *LungQuant v2*, respectively. The metrics are the vDSC and sDSC computed with 5 mm of tolerance, and the Mean Absolute Error (MAE) computed on the percentage of lung volume involved in the COVID-19 pneumonia

Version	Lung (vDSC)	Lung (sDSC)	Lesion (vDSC)	Lesion (sDSC)	MAE (%)
LungQuant1	0.95 ± 0.01	0.95 ± 0.02	0.66 ± 0.13	0.76± 0.18	4.2
LungQuant2	0.96 ± 0.01	0.97 ± 0.01	0.69 ± 0.08	0.83 ±0.07	2.0

As described in the Materials and Methods, at the end of the CT inference, we applied our algorithm for the separation of left and right lungs. Figure 6 shows some examples of the output of this procedure computed on three cases taken from the MosMed data set.

**Fig. 7:**
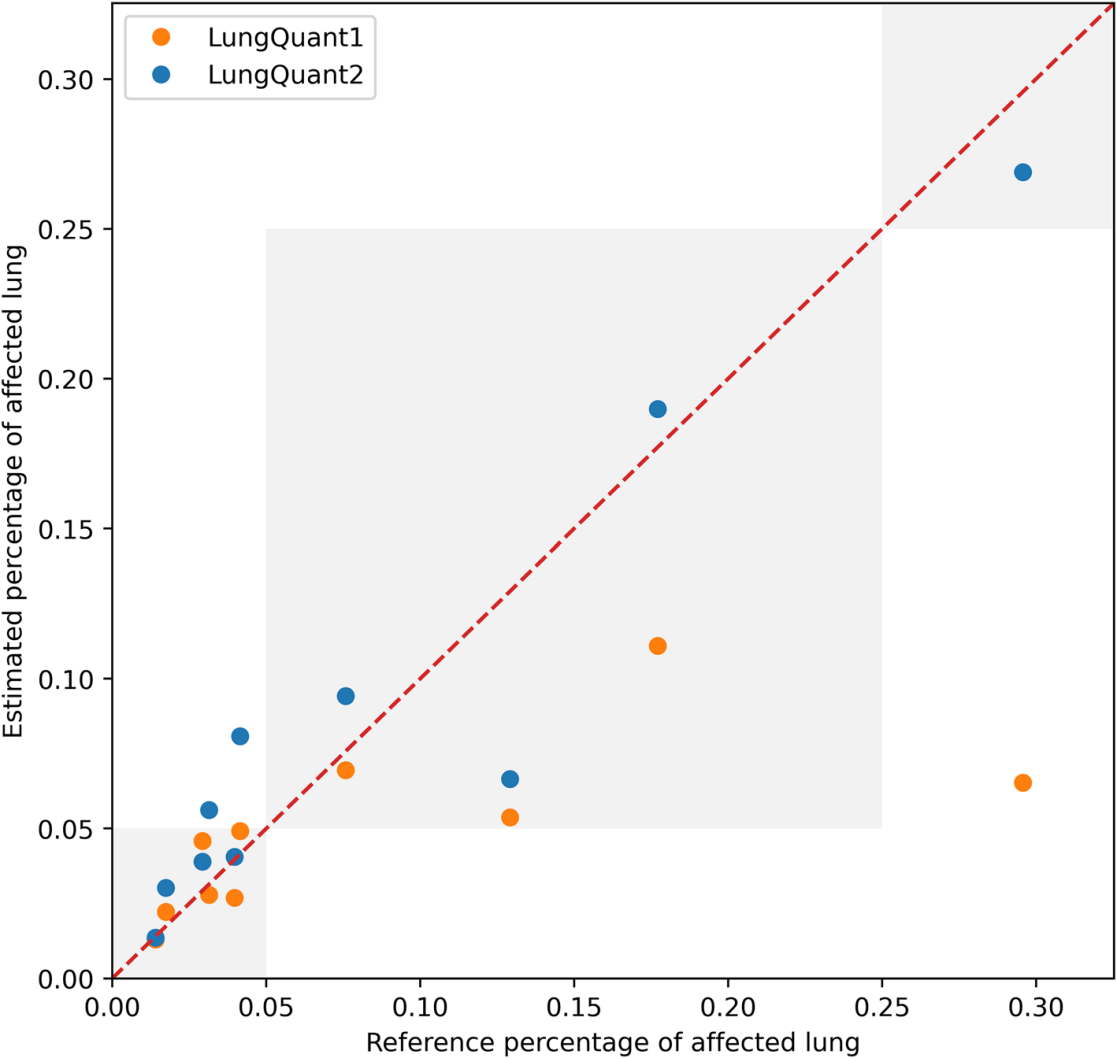
Estimated percentages *P* of affected lung volume versus the ground truth percentages, as obtained by the *LungQuant v2* system (blue circles). The performance obtained by the previously reported *LungQuant v1* system [30] is also shown as a reference (orange circles). The grey areas in the plot background guide the eye to recognize the CT-SS values to which each value of *P* is assigned (from left to right: CT-SS=1, CT-SS=2, CT-SS=3)

Lastly, we computed the volumes of the lungs and of the COVID-19 lesions and their ratio to obtain the percentage of involved lung volume and the CT-SS on the independent test set COVID-19-CT-Seg. We made a direct comparison of the performances of the *LungQuant v1* and *LungQuant v2* software versions in the correct CT-SS assessment. We reported the estimated percentage of affected lung volume versus the reference ones in Fig. 7. The capability of the system in providing a correct CT-SS is still satisfactory, since eight out of ten cases are correctly scored but lower with respect the first version of the software. However, the misclassification of one class at most occurs and the effect of the improvement is measurable in terms of the MAE that decreases from 4.2% of *LungQuant v1* to 2% of *LungQuant v2*, as reported in Table 2.

## Conclusions and discussion

In this work, we present an improved version of the *LungQuant* software (*LungQuant v2* [37]), a system trained to segment the lung parenchyma and the lesions related to COVID-19 pneumonia on lung CT scans. The algorithm, consisting in a sequence of three DNNs (BB-net, U-net$_1$ and U-net$_2$), provides the segmentation masks for lungs and lesions and the percentage of affected lung volume, also converted into the CT severity score. The segmentation strategy works as follows: (i) identification of the bounding box enclosing the lungs; (ii) lung segmentation; and (iii) COVID-19 lesion segmentation. The BB-net achieves a good performance, and the MSE on the test set is of order 10$^{-3}$. The contouring efficiency of *LungQuant* *v*2 reaches a vDSC (sDSC) equal to 0.96 ± 0.01 (0.97 ± 0.01) and 0.69 ± 0.08 (0.83 ± 0.07) for the lung and lesion segmentations, respectively. The performances of *LungQuant v2* have been directly compared to those previously presented for *LungQuant v1* [30]. The segmentation performances evaluated in terms of vDSC and sDSC of the two systems are fully consistent on the benchmark COVID-19-CT-Seg data sample of ten fully annotated CT scans. The advantage of *LungQuant v2* with respect to *LungQuant v1* is its improved capability in segmenting the lungs also in case the CT scans were either acquired or reconstructed with a small FOV. This is due to the BB-net prepended to the segmentation pipeline, which has been trained to recognize the bounding box enclosing the lungs on images with variable FOV, artificially generated though data augmentation. Adding this initial module to the analysis facilitates the learning process of the lung segmentation network (U-net$_1$), which receives only a subvolume of the chest image containing the lungs resampled to a standardized size.

As regard the lung segmentation task, the *LungQuant* performances compare well with those obtained by Ma et al. [29]. Furthermore, a limitation of the *LungQuant* *v*1 software was to be prone to underestimate the amount of lung involvement in more severe cases as visible in Fig. 7. This limitation in the system performance was due to the fact that most cases in the annotated data samples belong to low CT-SS classes. The introduction of a new term in the loss function of the U-net$_2$ of the *LungQuant* *v*2 version helped the system in generating a more linear response with case severity, as visible in Fig. 7 and demonstrated by the smaller MAE obtained. However, more populated and representative samples, which could allow balancing training examples according to the severity of radiological findings, would improve the U-net$_{2}$ capability to correctly segment larger lesions. Training ML systems on balanced datasets is a crucial point to obtain homogeneous performances that are independent from the severity of the disease. The current lack of a large dataset, fully representative of the underlying population, i.e. collected by paying attention to adequately represent all categories of disease severity, limits the possibility to carry out accurate training of AI-based models.

The possibility to access more populated and fully annotated data samples is fundamental to push the performance of image processing models based on DNNs. Learning from data where the characteristics of a heterogeneous population are adequately represented helps the DNN models to reach better and more homogeneous performances on previously unseen examples, i.e. to improve its generalization ability. As a final consideration, this segmentation and quantification work opens the way to lesion characterization studies. The segmentation of lungs and lesions related to COVID-19 pneumonia is a prerequisite to the extraction of radiomic features that can help to distinguish COVID-19 infection from other non-COVID related pneumonia and to develop predictive models of patients’ outcome. In this direction, the work by Fang et al. [28] developed an AI-based method to predict a severity score, which showed the remarkable performance of AUC = 0.813 in predicting the subjects’ intensive care unit admission. To evaluate the capability of our *LungQuant*2 system to enable the development of predictive models of disease progression and patients’ outcome, the availability of a fully annotated database with phenotypic and clinical information of patients is required. Lastly, the *LungQuant* segmentation software underwent a clinical validation made on 120 CT scans and its outputs have been compared to the visual assessments of CT images by 14 radiologists coming from five different Italian hospitals [38].

## Data Availability

This manuscript has associated data in a data repository. [Authors’ comment: Data repositories are properly reported in the bibliography with references to access and use them.]

## Footnotes

1 https://doi.org/10.15161/oar.it/76937.
